# Genetically Programmed Differences in Epidermal Host Defense between Psoriasis and Atopic Dermatitis Patients

**DOI:** 10.1371/journal.pone.0002301

**Published:** 2008-06-04

**Authors:** Patrick L. J. M. Zeeuwen, Gys J. de Jongh, Diana Rodijk-Olthuis, Marijke Kamsteeg, Renate M. Verhoosel, Michelle M. van Rossum, Pieter S. Hiemstra, Joost Schalkwijk

**Affiliations:** 1 Laboratory of Skin Biology and Experimental Dermatology, Nijmegen Centre for Molecular Life Sciences, Radboud University Nijmegen Medical Centre, Nijmegen, The Netherlands; 2 Department of Pulmonology, Leiden University Medical Centre, Leiden, The Netherlands; Massachusetts General Hospital and Harvard Medical School, United States of America

## Abstract

In the past decades, chronic inflammatory diseases such as psoriasis, atopic dermatitis, asthma, Crohn’s disease and celiac disease were generally regarded as immune-mediated conditions involving activated T-cells and proinflammatory cytokines produced by these cells. This paradigm has recently been challenged by the finding that mutations and polymorphisms in epithelium-expressed genes involved in physical barrier function or innate immunity, are risk factors of these conditions. We used a functional genomics approach to analyze cultured keratinocytes from patients with psoriasis or atopic dermatitis and healthy controls. First passage primary cells derived from non-lesional skin were stimulated with pro-inflammatory cytokines, and expression of a panel of 55 genes associated with epidermal differentiation and cutaneous inflammation was measured by quantitative PCR. A subset of these genes was analyzed at the protein level. Using cluster analysis and multivariate analysis of variance we identified groups of genes that were differentially expressed, and could, depending on the stimulus, provide a disease-specific gene expression signature. We found particularly large differences in expression levels of innate immunity genes between keratinocytes from psoriasis patients and atopic dermatitis patients. Our findings indicate that cell-autonomous differences exist between cultured keratinocytes of psoriasis and atopic dermatitis patients, which we interpret to be genetically determined. We hypothesize that polymorphisms of innate immunity genes both with signaling and effector functions are coadapted, each with balancing advantages and disadvantages. In the case of psoriasis, high expression levels of antimicrobial proteins genes putatively confer increased protection against microbial infection, but the biological cost could be a beneficial system gone awry, leading to overt inflammatory disease.

## Introduction

Psoriasis vulgaris and atopic dermatitis are two common chronic inflammatory skin diseases, characterized by various different clinical and histological features depending on the stage of the disease. Although both diseases are generally regarded as immune-mediated conditions, recent genetic studies have indicated the importance of abnormalities in epithelium-expressed genes as a primary cause. Loss of function alleles of the skin barrier protein filaggrin were found to be a major predisposing factor for atopic dermatitis[1], and we have recently demonstrated that a copy number polymorphism of a beta defensin gene cluster was associated with increased risk for psoriasis[2].

Lesional skin of patients with psoriasis or atopic dermatitis is heavily infiltrated with activated T cells that produce proinflammatory cytokines including those designated as Th1 cytokines (e.g. interferon-gamma (interferon-γ) and tumor necrosis factor alpha (TNF-α)) or Th2 cytokines (e.g. interleukin (IL)-4, IL-5 and IL-13). Psoriasis is generally regarded as a disease dominated by Th1 cytokines, whereas atopic dermatitis, particularly in active lesions, is driven by Th2 cytokines. Atopic dermatitis skin shows a high frequency of bacterial colonization and recurrent skin infections by bacterial, fungal, and viral pathogens. In contrast, a large epidemiological study on disease concomitance in psoriasis revealed that psoriasis patients have an increased resistance to bacterial and viral infections compared with controls and atopic dermatitis patients[3]. Several studies have shown that expression levels of antimicrobial proteins such as hBD-2, LL-37 and SLPI are significantly decreased in lesional atopic dermatitis skin compared with lesional psoriatic skin[4], [5]. It was speculated that a relative deficiency in expression of innate immunity genes in atopic dermatitis patients could account for the susceptibility to skin infection with *Staphylococcus aureus* [4]. In addition, microarray analysis on lesional skin of psoriasis and atopic dermatitis patients revealed a specific difference in the profile of expressed proinflammatory cytokines and chemokines[6]. These findings raised the question whether these differences are an acquired characteristic caused by extrinsic factors such as the inflammatory infiltrate and the cytokine environment, or alternatively could be driven by differences in genetic programming of epidermal keratinocytes in psoriasis and atopic dermatitis. Clearly, these mechanisms are not mutually exclusive. A few recent *in vitro* studies have shown that differences in the cytokine environment could be responsible for the observed differences in antimicrobial gene expression, as it was shown that IL-4, IL-13 and IL-10 downregulate defensin expression[7], [8].

As the epidermal inflammatory response of psoriasis and atopic dermatitis patients shows two opposite directions (i.e. high and low expression of host defense genes), the aim of the present study was to investigate if cell-autonomous differences exist between keratinocytes from psoriasis and atopic dermatitis patients. Our results show that the genetic programming of keratinocytes from psoriasis or atopic dermatitis patients is different between both diseases with respect to expression of genes involved in cutaneous inflammation and host defense.

## Results

**Figure 1:**
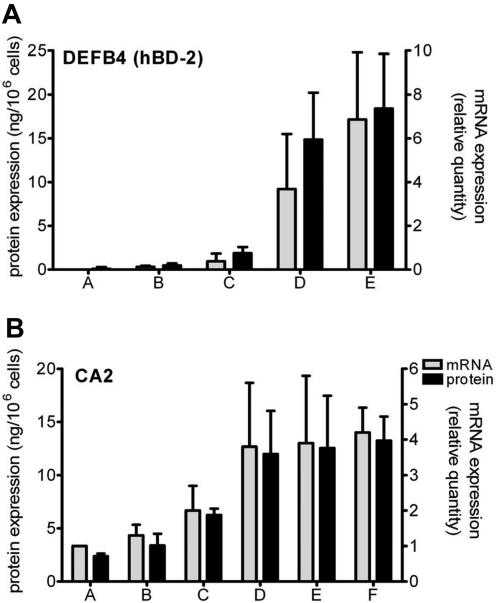
Cytokine-induced gene expression of DEFB4 and CA2 in cultured keratinocytes. (A) The Th1 cytokine mixture (IL-1α/TNF-α/interferon-γ) induces a dose-dependent increase of DEFB4 expression both for mRNA (grey bars) and protein (black bars); mean±SD of three cultures. Th1 cytokines concentrations used are in ng/ml (IL-1α and TNF-α) or U/ml (interferon-γ) respectively: A = no stimulus; B = 1.7 ng/1.7 ng/0.5 U; C = 5 ng/5 ng/1.7 U; D = 10 ng/10 ng/3.3 U; E = 30 ng/30 ng/10 U. (B) The Th2 cytokine mixture (IL-4/IL-13) induces a dose-dependent increase in mRNA and protein expression of the CA2 gene (mean±SD of five cultures). Cytokine concentrations are in ng/ml. A = no stimulus; B = 0.08 ng/0.08 ng; C = 0.4 ng/0.4 ng; D = 2 ng/2 ng; E = 10 ng/10 ng; and F = 50 ng/50 ng.

To create an *in vitro* model system to examine differences between keratinocytes from various diseases, we used a well-defined submerged keratinocyte culture model. First passage normal human keratinocytes were cultured in serum-free keratinocyte growth medium (KGM), and differentiation was induced by growth factor withdrawal, which causes the expression of differentiation-related proteins such as cytokeratin 10 and transglutaminase-1, as described before[9]. In this model that resembles normal human epidermis, disease-associated markers for epidermal activation (e.g. β-defensin-2 (hBD-2), psoriasin and elafin) are expressed at low to undetectable levels which makes it a suitable and sensitive model to study keratinocyte activation by inflammatory stimuli[10]. To mimic an inflammatory milieu as found in psoriasis, we stimulated normal human keratinocytes with a mixture of interferon-γ, TNF-α and IL-1α (pro-inflammatory cytokines; further referred to as Th1 cytokine mix). A combination of IL-4 and IL-13 was used as a Th2 cytokine mix, to resemble the atopic dermatitis cytokine microenvironment of active lesions. After 48 hours the culture supernatants were harvested and the mRNA was extracted from the cells for qPCR. It was found that Th1 cytokines induce a dose-dependent increase in expression of the psoriasis-associated gene DEFB4, which encodes the hBD-2 protein (Figure 1a). We found that Th2 cytokines did not induce expression of hBD-2 (not shown) but instead could dose-dependently induce the expression of carbonic anhydrase-2 (CA2) (Figure 1b), a gene previously found to be overexpressed in lesional atopic dermatitis skin[11] under control of Th2 cytokines[12]. Th1 cytokines did not induce expression of CA2 (not shown). These experiments exemplify how normal human keratinocytes are programmed to respond to Th1 or Th2 cytokines, with respect to these marker genes.

We used the model system, as described above, to expose cells to relevant stimuli that induce a disease-specific read-out *in vitro*. Therefore we tested 21 cell lines of primary keratinocytes derived from psoriasis patients, atopic dermatitis patients and healthy controls (cell cultures of uninvolved skin, from 7 donors for each group). mRNA from these cultures was used for quantitative analysis of a large panel of genes to examine if there were diagnosis-specific differences in expression profiles of resting or cytokine-stimulated keratinocytes. Genes to be analyzed were selected from the available literature on gene expression in lesional skin of psoriasis and atopic dermatitis[5], [6], [11], [13] and from a microarray study that we conducted on purified epidermal cells from lesional skin of psoriasis and atopic dermatitis patients. The experimental details and raw data of this study have been deposited in the NCBI Gene Expression Omnibus (GEO, http://www.ncbi.nlm.nih.gov/geo) and are accessible through GEO Series accession number GSE6601. The microarray study revealed 183 genes that showed significant differential expression between psoriasis and atopic dermatitis, and a number of genes from this list were selected for the present study (Table S1). The selected gene set comprised mainly genes involved in host defense and inflammation, such as antimicrobial proteins, cytokines and chemokines. We also included a number of genes that encode structural epidermal proteins such as cytokeratins 6, 10, 14 and 17, involucrin and connexin 43. Table S1 lists the primer sequences of 56 selected genes that were analyzed by qPCR on the 21 cell lines from patients and controls, cultured without stimulus or exposed to Th1 or Th2 cytokines. Table S2 contains the raw data of all qPCR analyses.

**Figure 2:**
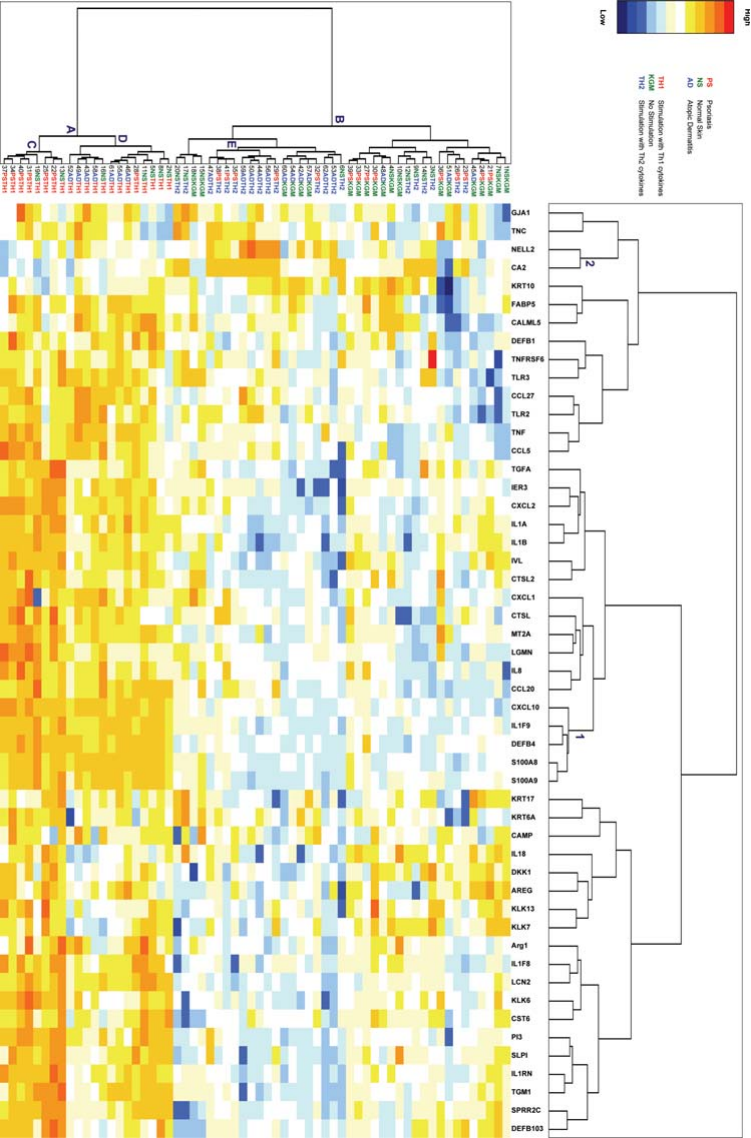
Two-way clustering of samples and expressed genes. Cluster analysis was performed on expression levels of 51 genes in 63 samples of cultured keratinocytes from healthy individuals (NS), psoriasis patients (PS) and atopic dermatitis patients (AD). Cells were left untreated (KGM) of stimulated with cytokines (Th1 and Th2). Only those genes that passed the test for false discovery rate (51 out of 55) were included in the analysis. qPCR data were subjected to Z-transformation and the Euclidian distance was used as a dissimilarity measure. Columns and rows were clustered by Ward’s amalgamation rule. Sorting in two dimensions reorganizes the data and generates an expression matrix depicted as a heat-map in which each cell was assigned a color corresponding to its normalized value. Gene clusters on the horizontal axis are numbered (1–2) as described in the text. Clusters of samples on the vertical axis are labeled A–E as described in the text.

We used a two-way clustering approach to analyze the structure of the data and to obtain a visual representation of the similarity between keratinocyte cultures, and genes that behave similarly across the different cell cultures (Figure 2). Clustering of the keratinocyte cultures clearly separates the Th1-stimulated cells (all samples under node A in Figure 2, referred to as cluster A) from the non-stimulated and Th2-stimulated cells, irrespective of their donor origin (all samples under node B, referred to as cluster B). This confirms and extends previous *in vitro* data indicating that cytokines like IL-1 and TNF-α strongly induce the expression of host defense genes such as members of the β-defensin family[14] and γ-interferon enhances this response. These findings support the notion that the cytokine environment *in vivo* is one of the factors that drive epidermal gene expression as seen in various diseases. Within cluster A, there is segregation of the psoriasis and atopic dermatitis cell lines with minimal overlap. Under node C, six out of seven psoriatic cell lines are clustered with two cell lines from normal individuals, whereas all atopic dermatitis cell lines are clustered under node D together with the remaining cell lines (one psoriasis and five normal individuals). Within cluster B (all non-stimulated and Th2 stimulated cell lines) clustering is less compact, although a homogeneous cluster of exclusively Th2 stimulated cells (under node E) can be discerned. This illustrates that keratinocytes derived from psoriasis patients can be discriminated from those of atopic dermatitis individuals on the basis of Th1-cytokine induced gene expression. On the horizontal axis of Figure 2 several clusters of putatively co-regulated genes are evident. Although the heat map reveals a large number of genes that show induction by Th1 cytokines, cluster 1 appears as a particularly compact cluster that contains a set of genes (CXCL10, IL1F9, DEFB4, S100A8, S100A9), which are strongly induced by Th1 cytokines in all diagnoses, compared to KGM and Th2 cytokines. Cluster 2, containing CA2 and NELL2, represents a small class of genes that are upregulated by Th2 cytokines. The reason that these two genes were included in our set was the reported *in vivo* overexpression in atopic dermatitis skin compared to psoriasis[11]. Our data basically show two things. Firstly, keratinocyte gene expression is strongly influenced by the respective cytokine environments regardless of the origin of the cells, and lends further support to the Th1/Th2 cytokine concept in psoriasis and atopic dermatitis. Secondly, the segregation of psoriasis and atopic dermatitis patients by cluster analysis points at cell-autonomous differences between keratinocytes of these patients.

**Figure 3:**
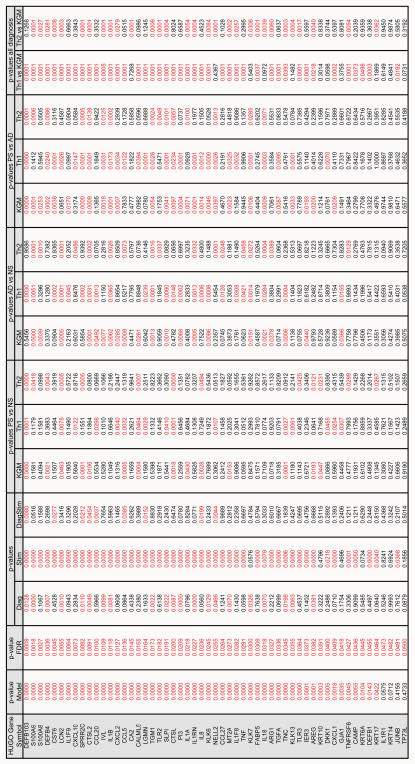
Summary of qPCR data. All genes that remained significant after FDR testing (third column), were used for factorial ANOVA. The p-values for the factor 'diagnosis' (normal skin (NS), psoriasis (PS) and atopic dermatitis (AD)), the factor 'stimulus' (KGM, Th1 and Th2) and the cross-effect (DiagStim) are in columns 4–6. The p-values for comparison between groups are given in columns 7–17, Post-hoc, Duncan's multiple range test. p-values <0.05 are marked in red.

**Figure 4:**
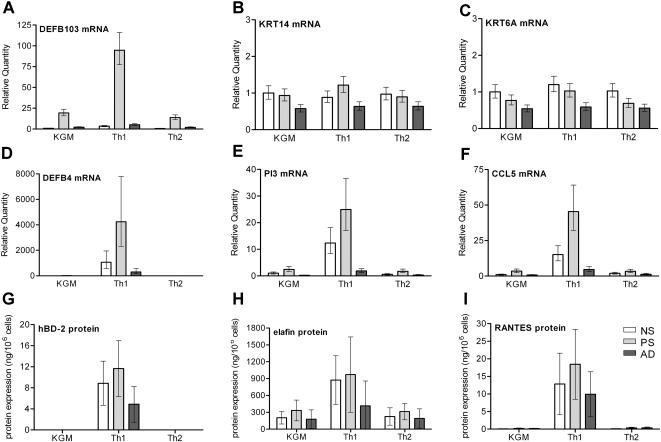
Graphical representation of mRNA and protein expression of selected genes. qPCR data of DEFB103, KRT14 and KRT6 (A–C); no significant effect of stimulus or diagnosis was found for expression of the cytokeratins KRT14 and KRT6, whereas the antimicrobial peptide DEFB103 (hBD-3) showed a significant effect both for stimulus and diagnosis. Statistical analysis was performed by ANOVA and post-hoc testing by Duncan's multiple range test, see Figure 3 for p-values, see Table S2 for raw data. qPCR data (D–F) and secreted protein levels (G–I) in the culture supernatant; the host defense genes DEFB4 (hBD-2) and PI3 (elafin), and the chemokine CCL5 (RANTES) show similar patterns of expression at the mRNA and protein level. A significant effect of stimulus and diagnosis was found by ANOVA and post-hoc testing by Duncan's multiple range test; see Figure 3 (qPCR) and Table S5 (protein assays) for p-values. qPCR data (A–F) are given in mean and standard error of seven cultures, protein data (G–I) in mean and standard deviation of seven cultures.

An exploratory statistical approach like cluster analysis is a useful strategy to analyze the structure of the data but does not allow testing for significance. Therefore the qPCR data (ΔCt values) were analyzed using a repeated factorial ANOVA design, combined with a recently described method for controlling the False Discovery Rate (FDR) instead of the older Family Wise Error Rate (FWER) by the classical Bonferroni or sequential Bonferroni tests like Holm's Step Down Test[15]. The two factors analyzed were ‘diagnosis’ (normal skin, psoriasis, atopic dermatitis) and ‘stimulation’ (KGM, Th1 cytokines, Th2 cytokines). A Duncan post-hoc test was performed to analyze all the single factors that remained significant after FDR testing. Figure 3 summarizes the p-values of the ANOVA, FDR and the post-hoc tests for all genes. This analysis showed that 48 genes were significantly regulated by the factor stimulation (column "Stim" in Figure 3). The last two columns of Figure 3 show the p-values of the individual genes for Th1 and Th2 stimulation. Although both Th1 and Th2 cytokines caused significant differences in gene expression, their effects are qualitatively and quantitatively different. Table S3 gives the least square means of the ΔCt values of all genes and the calculated fold stimulation. It is shown that Th1 cytokines have a strong stimulatory effect on expression of many genes involved in innate immunity and host defense, such as antimicrobial proteins (e.g. DEFB104, PI3, S100A8, S100A9), chemokines (e.g. CXCL10, CCL20, CCL27, CCL5 and IL8) and cytokines (e.g. IL1F9 and TNF). Th2 cytokines had either no effect or slight inhibitory effects on most genes, but increased the expression of NELL2, CA2 and TNC (more than 4-fold). The corresponding proteins of Th2-induced genes have diverse functions not normally associated with inflammation or host defense. As shown in Figure 2 we observed diagnosis-specific clustering of keratinocyte cell lines on the basis of gene expression data. The ANOVA data presented in Figure 3 indicate that expression levels of 21 genes were significantly regulated by the factor diagnosis. For 9 genes a significant cross-effect was found. Table S3 shows the fold difference between expression levels of genes, comparing the different diagnoses. For a substantial number of genes, the expression levels in psoriasis are considerably higher than in atopic dermatitis cells. These findings show that innate immunity genes, both encoding signaling molecules (e.g. IL8, IL1B, CXCL1) and effector molecules (DEFB4, DEFB103, PI3) are overexpressed by psoriasis keratinocytes compared to atopic dermatitis. Figures 4A, 4D, and 4E give a graphical representation of qPCR data from DEFB103, DEFB4 and PI3, as an example of innate immunity genes that are strongly induced by Th1 cytokines and show a significant diagnosis-specific response. For comparison, the expression of KRT14 and KRT6 is shown in Figures 4B and 4C as an example of two structural cytokeratins that are not cytokine-inducible and do not show diagnosis-specific differences.

The throughput and accuracy of analysis at the mRNA level is currently not matched by analysis at the protein level, although this would be desirable for obvious reasons. We selected a number of genes included in the study described above, for which immunoassays were available as ELISA or fluorescent bead assays (hBD-2, elafin, SLPI, CXCL8, RANTES and IP-10).We found stimulus-specific and diagnosis-specific differences for most proteins (factorial ANOVA, followed by post-hoc testing, see Table S4 for raw protein data, and Table S5 for p-values). All six genes selected for protein analysis were induced by Th1 cytokines, and for most proteins the expression by atopic dermatitis keratinocytes was significantly lower than for normal skin or psoriasis keratinocytes. A comparison of qPCR and protein data is shown for DEFB4 (hBD-2) (Figure 4D and G), PI3 (elafin) (Figure 4E and H) and CCL5 (RANTES) (Figure 4F and I) of the 63 cultures. The protein data largely confirm the mRNA data.

## Discussion

Our *in vitro* findings suggest that the observed high expression levels of innate immunity genes that have been reported in lesional skin of psoriasis patients compared to atopic dermatitis[4], [5], can be caused both by extrinsic factors (cytokines) and cell-autonomous (disease-specific) factors. We designed this study to use qPCR on a selected number of genes, many of which were for *a priori* reasons relevant to our question, based on known *in vivo* expression data. The restriction on the number of genes we analyzed, given a modest number of samples, circumvents methodological problems associated with other large scale expression studies such as microarray analysis that often preclude proper statistical analysis due to a high false discovery rate and huge family-wise errors. In addition, the accuracy and specificity of qPCR compared to microarray analysis further augments the power of this approach. To our knowledge there are no studies available that have performed large-scale analyses on keratinocytes from individuals with different diagnoses. Previous small-scale studies that have addressed *in vitro* differences between normal keratinocytes and/or keratinocytes from psoriasis and atopic dermatitis patients, did not detect significant cell-autonomous differences[16], [17]. A study on *in vitro* expression of a limited number of chemokines by cultured keratinocytes, using semi-quantitative PCR, suggested disease-specific differences between psoriasis and atopic dermatitis[18], although this concept was not supported in more recent studies[7], [19]. The failure to detect cell-autonomous differences could be explained by various methodological reasons such as experimental design, the genes selected for read-out, or the nature of the stimuli and the sensitivity of the culture system to detect differences. Cell-autonomous differences have previously been detected in cultured bronchial epithelial cells derived from patients with atopic asthma and healthy non-atopic controls[20]. A deficient response to rhinovirus was observed in cells from asthmatics, showing a decreased production of interferon-β, impaired apoptosis and increased virus replication. In atopic dermatitis, there is also evidence for increased sensitivity to infections, but this appeared to be secondary to exposure of keratinocytes to a Th2 milieu, resulting in suppression of expression of the antimicrobial peptide hCAP-18 (LL-37)[8]. In line with this, we observed that addition of Th2 cytokines to Th1 cytokines resulted in a marked suppression of the ability of Th1 cytokines to induce expression of hBD-2 and SLPI in keratinocytes from all subject groups (data not shown).

We interpret our findings in a way that differences exist in genetic programming of keratinocytes from psoriasis or atopic dermatitis patients with respect to expression of genes involved in cutaneous inflammation and host defense. We do not think that the observed differences are caused by 'simple' differences or polymorphisms in one or two genes. They are probably the outcome of a summation of many subtle polymorphisms, that would be undetectable by a genome-wide association study because of the very small relative risks associated with each factor. The summation of these genetic factors, which we would call 'the genetic network' would alter the basic 'setting' of the epidermal keratinocyte with respect to host defense or response to stress and infection.

Remarkably, these cell-autonomous differences were also noted when unstimulated, non-lesional cells of patient groups were compared, although the differences were most pronounced upon stimulation by Th1 cytokines. Based on qPCR analysis, ANOVA showed highly significant differences between diagnoses for the entire model and in many cases also for individual genes. Although the data set of the protein assays was substantially smaller than that of the qPCR assays, we did observe significant diagnosis-specific effects for 5 proteins (see Table S5). At the level of individual genes, cytokine-stimulated keratinocytes from psoriasis patients produced significantly higher levels of elafin and hBD-2 than keratinocytes from atopic dermatitis patients (see Figure 4).

Although we interpret the observed differences between psoriasis and atopic dermatitis keratinocytes to be genetically programmed, one should bear in mind that other mechanisms could be involved as well. A possible explanation for the differences in diagnosis-specific gene expression could be epigenetic mechanisms that are induced in the keratinocytes by the underlying disease. To date there is no evidence that this is the case, but it is an intriguing possibility that requires further investigation. An alternative explanation which has been coined repeatedly over the last several decades, is an occult viral infection in psoriasis, although this was never confirmed by independent studies[21].

Previous understanding of diseases such as psoriasis and atopic dermatitis has focused on mechanisms of the adaptive immune system, often with emphasis on the Th1-Th2 paradigm. Our present data, and findings from genetic association studies in atopic dermatitis[1], Crohn's disease[22], [23] and psoriasis[2] suggest that further understanding of innate immunity and barrier function of the epithelium is essential. We suggest that clusters of innate immunity genes, both with signaling and effector functions, are coadapted, each with balancing advantages and disadvantages. We interpret disease states, which are clinically defined as psoriasis or atopic dermatitis, as the pleiotropic effects of these coadapted polymorphisms. The ultimate outcome is reflected by activity or levels of expressed protein leading to functional consequences in physiology and, sometimes, a phenotypic manifestation known as disease. In the case of psoriasis, epidermal keratinocytes could have lower thresholds for expression of innate immunity genes (antimicrobials, chemokines), which would confer increased protection against microbial infection. There is evidence from epidemiological studies that the latter is indeed the case[3]. The biological cost of increased protection would be a beneficial system gone awry that leads to overt chronic inflammatory disease. The disease mechanism could involve excessive cytokine production and a genetically determined epidermal hypersensitivity to these factors derived from the local, possibly autoreactive, T-cell infiltrate. The causes of the cutaneous infiltration by T-cells and the nature of this increased spontaneous and cytokine-induced expression of host defense genes are currently unknown. The mechanisms of increased gene expression levels in psoriatic keratinocytes could be at the level of cell surface receptors, or more likely in the downstream signaling cascades. Speculatively, this could involve MAPkinase and/or NFκB signaling, as many of the genes overexpressed in psoriatic skin (e.g. DEFB4, PI3 and IL8) are regulated by these pathways[24]–[26]. The beta defensin cluster on chromosome 8p23 presents a special case of innate immunity genes associated with psoriasis, as their increased expression can be explained by three mechanism: increased copy number, Th1 cytokine stimulation of keratinocytes, and a cell-autonomous low threshold for cytokine stimulation. Also in atopic dermatitis the current emphasis is now moving away from an exclusive focus on adaptive immunity as the primary cause, towards local responses in the epithelium and quality of epidermal barrier function[1], [27]. With respect to expression of innate immune genes in atopic dermatitis epidermis, at least two distinct mechanisms could be operative. In (sub)acute lesions Th2 cytokines dominate over Th1 cytokines, which would prevent the induction of a strong host defense response, as we have indeed found when keratinocytes were exposed to mixtures of Th1 and Th2 cytokines (data not shown). Furthermore, we provide evidence for an additional mechanism by showing that atopic dermatitis keratinocytes are less responsive to the stimulatory effect of pro-inflammatory Th1 cytokines on expression of host defense mechanisms. Both mechanisms are in line with previous observations showing decreased expression of host defense proteins in atopic dermatitis skin[4], [5], which may explain the observed high frequency of infections in atopic dermatitis as compared to psoriasis[3]. Collectively, our data warrant a re-appraisal of the role of epidermal keratinocytes in inflammatory skin diseases[27], [28].

## Materials and Methods

### Microarray analysis

The microarray platform used was a printed 19 K oligonucleotide set (18,861 oligonucleotides representing 18,664 unique sequences) from Sigma-Genosys, Cambridge, UK). Detailed procedures for preparation of purified epidermis, RNA purification, linear RNA amplification, probe labeling, array printing, array hybridization and microarray analysis have been published elsewhere[5]. Experimental data on gene expression levels in lesional psoriasis and atopic dermatitis epidermal cells have been deposited, compliant with MIAME criteria, at http://www.ncbi.nlm.nih.gov/geo/ and are accessible through GEO Series accession number GSE6601.

### Cell culture

Primary human epidermal keratinocytes were cultured from skin biopsies of psoriasis patients (n = 7), atopic dermatitis patients (n = 7) and healthy volunteers (n = 7), following the Rheinwald-Green system[29], and stored in liquid nitrogen until use. Permission for these studies was obtained from the local medical ethics committee (Commissie Mensgebonden Onderzoek Arnhem-Nijmegen), and volunteers gave written informed consent. The study was conducted according to the Declaration of Helsinki principles. Biopsies were from trunk skin, and in the case of patients, biopsies were taken from distant uninvolved (non-lesional) skin of the trunk. All psoriasis patients had plaque-type psoriasis. Atopic dermatitis was diagnosed according to the Hanifin criteria, and included three intrinsic and four extrinsic type patients. All diagnoses were made by a dermatologist. Patient groups consisted of adult individuals aged 43±17 for psoriasis patients, 37±14 for atopic dermatitis patients and 31±13 for healthy controls (mean and SD). First-passage cells were cultured to confluency in keratinocyte growth medium (KGM), and induced to differentiate by growth factor depletion as described before[10]. Differentiating cell cultures were stimulated with Th1 cytokines (30 ng/ml IL-1α, 30 ng/ml TNF-α, 10 U/ml interferon-γ), Th2 cytokines (50 ng/ml IL-4 and 50 ng/ml IL-13), or left untreated (control). IL-1α, TNF-α, IL-4 and IL-13 were obtained from Peprotech and interferon-γ from HyCult Biotechnology. After 48 hrs the supernatant was collected and the cells were harvested for mRNA isolation.

### Quantitative real-time PCR

First-strand cDNA was generated from mRNA and the reverse transcriptase reaction products were used for quantitative real-time PCR, which was performed with the MyiQ Single-Colour Real-Time Detection System for quantification with Sybr Green and melting curve analysis (Bio-Rad) as previously described[30]. Primers were designed using Primer Express 1.0 Software (Applied Biosystems) and produced by Biolegio. Primer validation, qPCR reactions, and determination of relative mRNA expression were performed as previously described[5]. Expression of target genes was normalized to that of human ribosomal phosphoprotein P0 (RPLP0). This housekeeping gene was not found to be subject to regulation in keratinocyte cultures, irrespective of stimulation or diagnosis, and is more reliable than other reference genes such as ACTB (actin) or GAPDH (data not shown). Statistical analysis was performed as described below and in Text S1. For graphical representation of qPCR data (as in Figures 1 and 3) the method described by Livak[31] was used, and the mean expression level of non-stimulated (KGM) keratinocytes from normal skin (NS) was assigned the value 1. See Table S1 for primer sequences.

### Protein assays

Protein concentrations for IP-10 and RANTES were determined with the Bio-Plex fluorimetric bead assay (Bio-Rad), according to the manufacturer’s protocol. ELISA assays for elafin and SLPI were performed as described previously[32], [33]. ELISA for hBD-2 was performed using antisera against recombinant hBD-2 (Peprotech). An ELISA kit for the detection of CXCL8 was used in accordance to the protocol provided by the manufacturer (Biosource). CA2 protein levels were determined as described previously[12].

### Statistics

All data were analyzed with the Statistica software package version 7.0 (StatSoft Inc). All data and a detailed description of the statistical procedures are given as supplementary Tables S2 and S4, and Text S1.

## Supporting Information

Table S1 list of genes (approved gene symbols, protein names) and primers used for qPCR
(0.10 MB PDF)

Table S2 qPCR data (Ct values) of 56 genes for all cultures
(0.08 MB PDF)

Table S3 least square means of ΔCt values of each gene for all diagnoses and stimuli; fold increase of stimuli and diagnoses
(0.08 MB PDF)

Table S4 protein data on 6 genes for all cultures
(0.07 MB PDF)

Table S5 p-values of post-hoc test on protein data
(0.06 MB PDF)

Text S1 statistics and graphical representations
(0.01 MB RTF)
