# Supplementary material for: Genetically Programmed Differences in Epidermal Host Defense between Psoriasis and Atopic Dermatitis Patients
Source: PLoS One. 2008 Jun 4;3(6):e2301. doi: 10.1371/journal.pone.0002301 (PMC2409155; doi:10.1371/journal.pone.0002301)
Supplement: Table S1 — list of genes (approved gene symbols, protein names) and primers used for qPCR (0.10 MB PDF) [file pone.0002301.s001.pdf]

Table S1. Primers used for quantitative real-time PCR

HUGO Gene Symbol	ENSemble Transcript no.	Description Gene/Protein	Forward primer	Reverse primer	E*
AREG	ENST00000264487	amphiregulin	tggaagcagtaacatgcaaatgtc	ggctgctaatgcaatttttgataa	1.94
ARG1	ENST00000237310	arginase 1	acagtttggcaattggaagca	cacccagatgactccaagatcag	2.13
CA2	ENST00000285379	carbonic anhydrase II	aacaatggtcatgctttcaacg	tgtccatcaagtgaaccccag	2.00
CALML5	ENST00000315247	calmodulin-like 5, CLSP	ggttgacacggatggaaacg	aacctcggagatgagtttccttag	2.06
CAMP	ENST00000296435	cathelicidin, LL37	ccaggcccacgatggat	accagcccgtccttcttga	1.83
CCL20	ENST00000358813	SCYA20, MIP3-α	tggccaatgaaggctgtga	gatttgcgacacagacaactt	1.83
CCL27	ENST00000259631	CTACK	ccaggctttcgtgcttcac	ctttctctcttggtgctcaaacc	2.00
CCL5	ENST00000293272	RANTES	tctgcgctcctgcatctg	gggcaatgtaggcaaagca	1.90
CST6	ENST00000312134	cystatin M/E	tccgagacacgcacatcatc	ccatctccatcgtcaggaagtac	1.96
CTSL	ENST00000257498	cathepsin L	gttgctattgatgcaggtcatga	actgctacagtctggctcaaaataaa	1.86
CTSL2	ENST00000259470	cathepsin L2/V, CTSV	gctatggatgcaggccattc	acaccatgatccaggtttttgc	1.97
CXCL1	ENST00000296031	GRO1, SCYB1	gcggaaagcttgcctcaa	tcagcatcttttcgatgattttctt	2.00
CXCL10	ENST00000306602	SCYB10, IP10	ttcctgcaagccaattttgtc	tcttctcacccttctttttcattgt	1.99
CXCL2	ENST00000264492	GRO2, SCYB1, MIP2	cgcccatggttaagaaaatca	ccttctggtcagttggatttgc	1.94
DEFB1	ENST00000297439	hBD-1, defensin β1	gatgcctcttccaggtgttttt	ggatgacatatggctccactctt	2.00
DEFB103	ENST00000318124	hBD-3, defensin β3	gtgaagcctagcagctatgaggat	tgattcctccatgacctggaa	2.06
DEFB4	ENST00000318157	hBD-2, defensin β2	gatgcctcttccaggtgttttt	ggatgacatatggctccactctt	1.97
DKK1	ENST00000224974	dickkopf, xenopus homolog	atcatagcaccttggatgggtatt	cttcttgtcctttggtgtgatacattt	2.13
FABP5	ENST00000297258	fatty acid binding protein 5	accctgggagagaagtttgaaga	tgtaaagttgcagacagtctgagtttt	2.20
FAS	ENST00000355740	TNFRSF6, FAS antigen	tgcagaagatgtagattgtgtgatga	gggtccgggtgcagtttatt	2.06
GJA1	ENST00000282561	connexin 43	caatcacttggcgtgacttca	accttgtcaaggagtttgcctaa	1.83
IER3	ENST00000259874	immediate early response 3	tcctgttttgtctccccttacg	tcaggatctggcagaagacgat	2.13
IL18	ENST00000280357	interleukin 18, IL-18	atcgcttcctctcgcaacaa	cttctactggttcagcagccatct	1.89
IL1A	ENST00000263339	interleukin 1α, IL-1α	cgccaatgactcagaggaaga	agggcgtcattcaggatgaa	2.20
IL1B	ENST00000263341	interleukin 1β, IL-1β	aatctgtacctgtcctgcgtgtt	tgggtaatttttgggatctacactct	2.20
IL1F8	ENST00000259213	IL1 family member 8, IL-1F8	accaccatctgatctatcttgttctct	gtgctgcctcccgttgtg	1.89
IL1F9	ENST00000259205	IL1 family member 9, Il-1F9	aggaagggccgtctatcaatc	gaactgccacaaggttctgac	2.06
IL1R1	ENST00000233946	interleukin 1 receptor, type 1	cctgctatgattttctcccaataaa	aacacaaaaatatcacagtcagaggtagac	1.83
IL1RN	ENST00000354115	IL-1 receptor antagonist, IL-1RA	cagctggaggcagttaacatcac	ccactgtctgagcggatgaa	2.00
IL8	ENST00000307407	interleukin 8, IL-8, CXCL8	cttggcagccttcctgattt	ttctttagcactccttggcaaaa	2.06
IVL	ENST00000295365	involucrin	acttatttcgggtccgctaggt	gagacatgtagagggacagagtcaag	1.89
JUNB	ENST00000302754	oncogene JUN-B	cgcccggatgtgcacta	cggccgtatcccgtagct	1.89
KLK13	ENST00000156476	kallikrein 13	gtgaattaccccaaaactctacaatgt	agacttgacgacactcctcatctg	2.06
KLK6	ENST00000310157	kallikrein 6	gagcggccatgaagaagct	tccgccatgcaccaactta	2.13
KLK7	ENST00000304045	kallikrein 7, SCCE	gcatccccgactccaagaa	cagggtacctctgcacaccaa	1.89
KRT10	ENST00000269576	keratin 10, CK10	tggttcaatgaaaagagcaagga	gggattgtttcaaggccagtt	1.91
KRT14	ENST00000167586	Keratin 14, CK14	ggcctgctgagatcaaagactac	cactgtggctgtgagaatcttgtt	1.91
KRT17	ENST00000326333	keratin 17, CK17	catgcaggccttggagataga	cacgcagtagcggttctctgt	2.00
KRT6A	ENST00000252250	keratin 6A, CK6A	agagaatgaatttgtgactctgaagaag	tacaaggctctcaggaagttgatct	2.08
LCN2	ENST00000277480	lipocalin 2, NGAL	caatgtcacctccgtcctgttta	ctggcaacctggaacaaaagtc	2.13
LGMN	ENST00000334869	legumain, AEP, PRSC1	tgcagtatggaaacaaaacaatctc	gggtgaggtcaaggtgtgtga	1.85
MT2A	ENST00000245185	methallothionein 2A	gcccagggctgcatctg	tttgtggaagtcgcgttcttta	2.01
NELL2	ENST00000333837	Nel-like2	taagggtataatgcaagatgtccaatt	agatctgggcactgagcaataaa	2.06
PI3	ENST00000243924	SKALP, elafin	catgagggccagcagctt	tttaacaggaactcccgtgaca	2.00
RPLP0	ENST00000228306	ribosomal phospoprotein P0, hARP	caccattgaaatcctgagtgatgt	tgaccagcccaaaggagaag	2.00
S100A8	ENST00000271846	calgranulin A, MRP8	ccgagtgtcctcagtatatcaggaa	acgcccatctttatcaccagaat	1.97
S100A9	ENST00000295382	calgranulin B, MRP14	tgtggctcctcggctttg	gcgttccagctgcgacat	2.20
SLPI	ENST00000338380	ALP, HUSI	ttcccctgtgaaagcttgattc	gatatcagtggtggagccaagtc	1.95
SPRR2C	ENST00000290702	small proline-rich protein 2C	acccctgaagtgccctgag	gggaaggtgtcaccagaggata	2.00
TGFA	ENST00000295400	transforming growth factor α	gccatttaatggcaatggtagtct	cacagggagcttgcagagat	2.13
TGM1	ENST00000206765	transglutaminase 1, TGase-1, TGK	cccccgcaatgagatctaca	atcctcatggtccacgtacaca	1.89
TLR2	ENST00000260010	toll-like receptor 2	tccgtctttttgatgagaacaatg	actccaggtaggtcttggtgttca	1.89
TLR3	ENST00000296795	toll-like receptor 3	tttgcgaagaggaatgtttaaatct	cacctatccgttctttctgaactg	1.89
TNC	ENST00000350763	tenascin C	aagtgaacctgtctcagggtcatt	gctgtcaccaggccagatg	2.20
TNF	ENST00000229681	tumor necrosis factor α	tcttctcgaaccccgagtga	cctctgatggcaccaccag	2.00
TP73L	ENST00000354600	P63 deltaN isoform	caatgcccagactcaatttagtga	tgctggtccatgctgttcag	1.83

*E is efficiency as fold increase in fluorescence per PCR cycle
