# Supplementary material for: Genetically Programmed Differences in Epidermal Host Defense between Psoriasis and Atopic Dermatitis Patients
Source: PLoS One. 2008 Jun 4;3(6):e2301. doi: 10.1371/journal.pone.0002301 (PMC2409155; doi:10.1371/journal.pone.0002301)
Supplement: Table S2 — qPCR data (Ct values) of 56 genes for all cultures (0.08 MB PDF) [file pone.0002301.s002.pdf]

Table S2. qPCR data (Ct values) of 56 genes for 21 cell lines and 3 culture conditions

Two batches of cDNA were used, as indicated between brackets following each gene name. RPLP0 was used as a reference gene. RPLP0 (1) was used for normalization of batch 1 and RPLP0 (2) was used for normalization of batch 2.

See Table S1 for gene symbols. NS = normal skin, PS = psoriasis, AD = atopic dermatitis

n/a = not analyzed

DIAG	STIM	RPLP0 (1)	RPLP0 (2)	AREG (1)	ARG1 (2)	CA2 (2)	CALML5 (2)	CAMP (1)	CCL20 (2)	CCL27 (2)	CCL5 (1)	CST6 (1)	CTSL (1)	CTSL2 (1)	CXCL1 (2)	CXCL10 (1)
NS1	KGM	17.80	17.91	21.73	31.96	19.68	27.47	31.27	32.16	31.63	31.97	22.15	27.09	22.29	30.45	34.31
NS1	TH1	18.48	18.56	23.68	31.01	20.12	27.11	32.01	27.08	29.75	30.56	21.42	28.02	22.68	28.03	29.71
NS1	TH2	18.30	17.22	22.83	30.48	17.59	26.78	33.34	31.74	30.10	30.67	23.40	28.17	24.01	29.68	33.90
NS2	KGM	18.19	18.66	24.63	31.99	20.76	24.88	32.70	31.04	31.20	32.43	23.45	27.06	21.59	31.06	33.07
NS2	TH1	18.63	19.15	23.93	30.86	21.09	24.57	31.70	26.55	27.69	28.86	22.34	27.63	22.34	28.26	27.62
NS2	TH2	18.91	17.20	27.39	30.84	17.63	24.00	34.95	30.06	28.92	33.93	24.66	28.82	23.90	30.23	34.79
NS3	KGM	16.82	17.09	21.62	30.45	19.88	27.00	29.97	27.57	33.08	31.79	21.08	26.84	20.60	27.02	35.77
NS3	TH1	17.54	17.96	24.11	31.12	20.67	25.58	31.12	25.87	29.84	27.66	21.61	26.63	21.65	27.94	26.70
NS3	TH2	17.05	17.58	24.90	31.37	18.18	25.25	31.90	29.60	30.46	29.79	22.91	26.46	22.52	29.26	33.71
NS4	KGM	18.81	19.81	25.27	31.09	22.04	25.82	33.06	32.96	32.51	32.69	24.11	29.13	23.06	32.21	n/a
NS4	TH1	18.79	18.88	23.89	29.08	21.17	24.63	31.96	27.27	29.90	28.59	21.94	27.81	21.92	26.39	29.00
NS4	TH2	17.28	18.20	23.96	31.42	18.79	25.33	31.85	32.99	31.81	29.97	22.96	27.65	22.79	30.78	32.40
NS5	TH1	18.58	18.62	23.16	29.03	21.42	27.72	31.56	26.79	28.78	27.75	21.54	27.01	21.50	25.77	27.65
NS5	TH2	17.07	17.64	23.53	30.56	18.25	27.69	31.25	31.04	30.74	27.87	22.90	27.01	22.31	28.72	32.96
NS6	KGM	17.75	18.36	24.79	32.01	21.80	26.24	29.87	26.98	29.54	28.68	26.57	26.53	20.73	26.13	30.87
NS6	TH1	18.18	18.64	24.16	33.39	21.98	26.89	30.91	25.70	27.71	25.67	25.35	26.72	21.89	23.68	25.92
NS6	TH2	18.34	18.79	24.95	32.67	19.41	27.40	31.71	29.17	31.24	29.33	27.94	27.26	22.84	27.28	30.89
NS7	KGM	18.00	18.20	24.48	30.40	21.40	24.94	34.02	30.03	32.44	30.62	26.09	26.96	21.29	23.06	34.17
NS7	TH1	18.74	19.07	23.64	32.00	23.20	25.10	31.96	24.77	28.40	25.62	24.35	26.44	21.77	34.65	26.02
NS7	TH2	17.63	17.87	24.44	33.39	18.66	25.50	34.37	28.36	31.85	29.63	26.36	26.05	22.48	26.44	31.48
PS1	KGM	16.62	17.12	21.27	32.00	19.33	27.98	31.96	30.57	30.08	29.44	23.46	25.48	21.85	28.76	32.97
PS1	TH1	17.81	18.16	21.79	29.62	20.24	26.75	34.00	27.06	27.76	27.28	22.04	25.02	20.77	24.64	26.94
PS1	TH2	16.32	16.98	21.84	31.79	17.29	28.11	30.39	30.64	29.62	29.37	23.82	24.89	22.69	28.54	32.32
PS2	KGM	16.40	16.70	20.82	31.10	20.07	27.88	32.36	29.86	32.19	28.77	22.82	25.50	21.90	27.48	33.90
PS2	TH1	17.68	17.92	20.94	30.39	21.46	28.11	32.18	26.95	30.12	27.76	21.88	25.96	21.37	27.11	26.46
PS2	TH2	16.17	16.68	21.11	31.66	18.11	28.57	30.93	30.73	30.37	29.25	22.83	25.08	22.30	27.34	32.99
PS3	KGM	16.85	17.19	22.87	30.33	19.81	24.10	31.97	27.65	27.99	28.58	22.59	25.54	21.80	24.90	29.00
PS3	TH1	17.11	17.70	22.22	29.61	20.51	23.63	30.97	25.24	27.27	25.58	21.71	25.63	21.62	26.19	25.05
PS3	TH2	16.72	17.09	23.01	31.99	17.49	25.31	32.59	25.70	26.54	26.05	23.20	25.71	22.58	28.88	29.72
PS4	KGM	16.98	17.69	21.21	30.33	20.52	26.24	29.40	29.50	30.64	29.90	20.87	26.37	19.99	28.15	35.25
PS4	TH1	18.00	18.18	23.98	28.16	20.69	25.14	31.15	26.30	24.96	25.91	20.39	26.41	20.08	21.93	24.31
PS4	TH2	17.44	17.97	23.01	31.44	n/a	28.18	30.21	31.00	31.14	30.62	21.91	26.66	22.34	29.14	34.39
PS5	KGM	17.44	17.88	22.11	31.46	20.81	26.41	32.63	28.95	30.16	29.07	23.14	26.06	21.73	28.31	31.19
PS5	TH1	17.88	18.02	22.25	30.32	20.96	26.50	29.73	27.00	26.61	24.72	22.17	25.59	20.97	24.69	23.87
PS5	TH2	17.15	17.81	22.25	31.77	18.39	26.87	30.83	27.40	29.66	27.67	23.58	26.10	22.32	27.81	32.33
PS6	KGM	17.39	18.09	23.16	31.60	22.50	27.76	30.87	32.07	30.88	28.17	22.48	25.89	19.90	25.15	34.73
PS6	TH1	18.04	18.19	22.00	31.91	22.04	27.08	31.89	28.17	28.03	23.90	22.92	25.92	20.87	24.03	23.31
PS6	TH2	17.50	17.59	23.12	31.97	18.13	26.17	31.23	30.60	30.07	28.67	23.91	25.44	22.36	28.54	31.15
PS7	KGM	17.35	17.77	22.41	32.28	20.53	26.71	32.03	28.30	28.97	27.20	23.15	25.51	22.14	25.60	32.14
PS7	TH1	18.45	18.61	23.74	32.08	21.91	25.64	31.74	26.65	26.99	24.90	22.10	25.73	22.00	23.86	26.11
PS7	TH2	17.69	17.91	24.15	32.91	18.16	26.11	32.37	27.52	29.01	28.68	24.65	25.82	23.48	23.76	32.38
AD1	KGM	17.24	17.61	23.96	30.74	20.29	25.92	31.42	30.89	30.26	28.88	23.49	26.57	22.39	30.71	33.70
AD1	TH1	17.90	18.28	23.03	30.62	20.63	24.81	31.14	27.82	25.75	26.61	22.89	26.25	21.75	24.79	26.85
AD1	TH2	17.06	17.84	24.16	32.33	18.10	25.96	31.12	29.29	28.73	27.99	23.09	26.28	23.13	29.09	n/a
AD2	KGM	17.24	18.05	21.61	32.26	21.15	27.39	31.11	30.86	31.75	28.77	23.57	26.11	22.30	29.99	34.88
AD2	TH1	17.63	18.36	21.43	30.23	21.14	26.03	31.58	27.91	27.19	27.14	21.86	25.91	21.57	25.01	26.88
AD2	TH2	17.26	17.56	22.70	31.52	18.23	27.15	31.84	29.87	30.22	27.72	23.65	26.33	22.99	26.92	34.73
AD3	KGM	17.19	17.34	23.47	29.73	19.48	23.82	30.98	28.29	28.15	26.91	21.79	26.60	22.32	28.99	32.03
AD3	TH1	17.84	18.53	23.72	29.74	20.80	24.23	30.15	27.25	25.52	25.66	22.79	25.88	22.58	25.22	26.78
AD3	TH2	17.27	17.71	24.07	31.64	17.96	25.05	30.61	28.13	27.24	27.04	21.69	26.61	22.89	28.83	33.13
AD4	KGM	16.60	17.03	22.25	31.47	21.73	28.86	29.83	31.52	29.83	28.70	24.68	26.20	21.75	30.43	n/a
AD4	TH1	17.31	17.82	22.80	29.87	21.65	26.45	31.19	27.98	27.90	26.05	24.71	26.12	21.85	26.20	26.64
AD4	TH2	17.21	17.65	22.25	33.02	18.47	27.76	31.79	29.60	32.52	29.05	26.02	26.49	24.31	31.68	34.24
AD5	KGM	17.64	17.82	23.34	33.03	20.22	25.21	32.63	30.05	29.72	30.51	23.89	26.82	22.92	30.56	33.91
AD5	TH1	17.61	17.93	21.91	31.13	20.52	24.65	31.33	26.50	27.61	26.93	23.08	25.77	21.94	27.29	26.55
AD5	TH2	17.23	17.62	23.34	33.62	18.26	25.77	32.58	30.03	29.71	27.59	23.65	26.58	23.19	29.70	33.46
AD6	KGM	17.17	17.82	23.15	32.24	20.42	26.49	30.42	30.70	29.62	29.56	24.14	26.00	21.73	27.83	32.81
AD6	TH1	17.81	18.43	22.81	33.02	20.77	25.88	34.10	27.73	26.80	27.26	23.45	25.77	21.78	25.30	25.64
AD6	TH2	17.20	17.85	22.55	31.95	18.25	26.82	31.53	28.77	24.93	28.55	24.34	25.94	23.26	28.75	32.74
AD7	KGM	17.11	17.75	24.05	31.74	n/a	25.95	32.14	30.86	29.48	29.30	23.99	26.66	22.82	30.89	34.42
AD7	TH1	17.77	17.82	22.59	30.81	n/a	25.48	31.71	27.01	26.12	27.89	23.04	26.25	22.36	25.21	27.33
AD7	TH2	17.06	17.38	24.31	32.81	n/a	27.34	31.87	29.72	30.06	28.89	24.77	26.47	23.64	29.11	34.51

DIAG	STIM	CXCL2 (1)	DEFB1 (1)	DEFB103 (1)	DEFB4 (1)	DKK1 (1)	FABP5 (1)	GJA1 (1)	IER3 (1)	IL18 (2)	IL1A (1)	IL1B (1)	IL1F8 (1)	IL1F9 (1)	IL1R1 (1)	IL1RN (1)
NS1	KGM	30.56	25.60	24.61	30.75	26.74	19.90	28.40	31.71	22.40	25.55	28.78	35.12	31.91	31.29	25.37
NS1	TH1	31.15	27.22	23.70	22.99	28.58	21.57	28.50	30.93	22.92	25.59	28.73	34.60	27.34	33.42	24.96
NS1	TH2	31.24	25.18	25.74	31.78	25.76	21.71	27.01	30.30	22.22	26.26	30.32	32.81	31.52	30.99	n/a
NS2	KGM	32.51	24.95	28.15	32.31	27.15	19.95	24.99	29.99	23.35	26.80	28.29	32.59	32.48	29.35	25.04
NS2	TH1	29.44	24.88	27.22	20.61	27.74	20.57	25.91	29.32	24.60	25.21	26.63	31.57	26.06	36.29	24.68
NS2	TH2	35.13	28.04	29.12	34.19	30.92	21.98	27.73	35.60	22.87	29.06	32.33	34.21	34.54	31.90	28.41
NS3	KGM	29.67	24.85	20.67	28.40	27.00	19.74	26.69	29.76	21.05	24.24	25.61	32.29	28.15	30.87	23.91
NS3	TH1	29.16	24.69	20.69	19.65	27.87	19.70	26.79	30.32	22.70	24.29	26.28	30.53	24.94	30.38	24.68
NS3	TH2	32.12	25.34	22.22	30.10	28.62	20.21	26.49	31.08	22.06	26.64	30.45	32.07	29.20	29.68	25.02
NS4	KGM	32.29	28.08	27.70	33.98	28.67	21.08	26.46	31.41	23.33	28.01	32.26	32.49	33.37	31.64	26.99
NS4	TH1	28.63	26.14	25.81	22.53	26.88	21.04	27.50	29.97	23.60	25.07	27.51	30.68	25.67	32.16	24.76
NS4	TH2	31.71	25.39	26.76	33.74	26.75	21.06	26.26	30.73	22.75	27.60	34.86	31.82	n/a	30.09	25.31
NS5	TH1	27.85	25.99	22.11	21.48	27.28	20.80	27.41	27.72	23.08	23.33	25.44	30.39	24.85	30.73	23.67
NS5	TH2	31.35	26.03	23.25	30.76	25.71	21.37	25.23	30.00	22.00	25.39	30.76	32.08	30.06	30.63	25.07
NS6	KGM	28.80	25.60	28.63	25.77	28.81	20.94	25.29	29.82	23.67	25.90	26.93	33.36	27.87	30.55	25.30
NS6	TH1	27.27	25.60	27.80	20.79	28.92	20.63	24.67	29.73	23.73	25.48	25.34	32.35	24.93	30.39	25.31
NS6	TH2	29.25	26.50	29.12	29.77	29.36	21.64	22.72	30.49	22.43	26.85	28.83	35.76	28.95	30.41	25.60
NS7	KGM	29.92	26.58	29.37	32.36	31.70	20.08	23.96	29.71	n/a	26.59	28.47	33.63	28.84	31.61	25.74
NS7	TH1	27.78	26.74	26.15	19.35	30.05	20.52	25.26	28.76	23.84	23.69	24.26	31.81	24.53	31.69	24.71
NS7	TH2	29.80	26.36	28.57	32.73	29.28	20.72	22.87	29.45	22.67	24.70	26.72	36.37	28.28	30.28	25.97
PS1	KGM	28.67	25.60	21.87	28.21	25.74	19.55	25.08	29.24	21.51	23.31	25.80	32.04	28.74	31.43	23.59
PS1	TH1	26.01	n/a	19.85	19.90	25.63	19.86	25.18	27.55	22.20	22.57	24.93	29.44	24.67	31.62	22.63
PS1	TH2	30.45	24.98	21.10	27.87	26.39	19.85	25.08	29.62	21.76	23.69	28.06	34.03	29.50	30.78	25.16
PS2	KGM	28.13	25.35	21.37	26.60	25.67	19.25	23.26	28.17	21.36	23.07	25.46	33.45	28.76	31.22	23.95
PS2	TH1	25.41	25.14	20.71	19.74	25.71	20.75	25.92	27.47	21.71	23.53	25.39	31.38	26.75	33.12	23.55
PS2	TH2	28.39	25.72	21.66	26.83	25.87	20.14	22.15	28.11	21.59	23.20	27.25	33.52	29.04	31.02	25.00
PS3	KGM	29.96	24.79	23.22	20.84	27.97	18.21	23.92	29.35	21.68	24.62	27.21	31.60	27.10	29.34	24.06
PS3	TH1	28.17	23.78	22.08	20.91	27.27	19.00	23.34	28.55	22.53	24.04	26.87	29.00	25.52	30.79	23.62
PS3	TH2	30.31	24.38	24.04	28.73	28.23	18.99	23.92	28.44	22.46	26.26	32.23	31.59	28.73	30.00	24.42
PS4	KGM	30.17	24.68	24.75	29.47	25.23	19.77	24.40	30.05	21.93	25.05	26.65	30.98	29.68	29.03	23.73
PS4	TH1	27.95	25.72	22.59	20.59	26.39	20.37	23.40	28.75	22.32	24.73	25.90	31.90	25.90	31.57	23.83
PS4	TH2	32.06	26.39	25.68	30.57	27.01	20.81	24.58	32.98	22.97	25.94	30.01	32.70	30.57	29.60	25.78
PS5	KGM	30.03	25.51	23.88	29.21	27.42	20.12	24.23	29.72	21.80	25.14	26.86	32.77	29.03	31.40	25.46
PS5	TH1	26.66	24.06	21.89	19.10	26.13	19.23	25.58	27.98	22.48	23.50	24.95	31.83	24.79	31.48	23.79
PS5	TH2	30.03	25.82	23.74	28.22	27.24	20.76	24.66	28.92	22.38	25.43	29.34	36.16	28.43	31.39	25.86
PS6	KGM	28.79	26.42	22.88	27.34	27.39	21.89	26.39	29.28	22.32	25.11	26.10	32.54	30.65	32.38	24.82
PS6	TH1	26.38	25.47	22.35	19.57	26.10	20.94	26.25	29.37	23.32	23.84	24.86	30.27	25.83	31.51	23.58
PS6	TH2	30.95	26.64	24.61	27.63	26.99	21.33	24.05	30.16	22.33	24.70	26.36	32.57	28.82	32.04	26.01
PS7	KGM	28.60	24.88	23.68	26.51	26.92	19.73	25.04	29.10	22.07	25.23	27.22	32.67	28.34	29.36	24.69
PS7	TH1	26.77	25.64	22.12	18.40	28.46	20.20	22.43	28.42	23.10	24.65	25.43	30.16	25.61	29.72	24.64
PS7	TH2	28.78	27.05	24.18	28.62	29.80	20.89	25.64	29.85	22.75	26.76	29.69	34.68	29.75	30.12	26.10
AD1	KGM	31.67	25.04	25.73	29.93	29.66	19.60	24.17	33.50	23.95	26.39	30.17	33.43	31.17	30.81	26.12
AD1	TH1	28.26	25.85	25.02	22.85	28.45	19.63	23.04	30.59	24.24	24.62	27.88	32.69	27.47	31.84	25.99
AD1	TH2	31.61	26.01	25.20	29.81	28.05	20.03	22.92	30.72	24.20	27.35	33.49	35.44	32.11	31.74	26.24
AD2	KGM	29.39	26.72	25.03	28.82	25.96	19.65	23.99	29.97	21.97	24.51	27.32	34.03	29.26	30.71	25.33
AD2	TH1	27.02	25.44	23.63	20.71	26.34	19.35	23.41	27.85	22.64	22.74	25.06	31.64	25.32	30.93	24.05
AD2	TH2	29.38	24.64	24.41	29.90	26.40	20.72	24.21	28.64	22.01	25.30	30.74	33.54	28.73	31.13	25.50
AD3	KGM	31.18	n/a	26.28	31.50	26.11	19.03	24.49	30.09	22.56	26.56	30.36	32.73	30.10	30.24	25.57
AD3	TH1	28.15	25.94	25.90	19.90	29.26	19.38	24.64	29.29	24.16	26.14	28.44	31.84	26.53	30.10	25.18
AD3	TH2	31.63	25.86	26.17	31.75	27.46	19.92	23.64	29.81	23.33	27.27	31.99	32.43	30.30	30.67	25.99
AD4	KGM	29.61	25.95	23.38	29.95	25.73	21.52	22.61	29.44	22.37	25.18	27.08	33.35	30.59	32.57	25.59
AD4	TH1	28.78	25.91	24.34	21.29	27.95	20.30	24.17	29.75	23.57	24.98	27.06	32.57	26.27	32.45	25.54
AD4	TH2	30.49	26.58	25.72	30.64	27.68	20.69	26.32	30.58	23.68	25.66	30.67	34.51	29.61	31.33	26.09
AD5	KGM	31.39	26.20	25.75	31.45	29.86	19.17	25.01	31.38	22.57	26.40	31.58	32.88	30.89	30.78	26.12
AD5	TH1	27.83	24.46	24.02	21.62	27.24	19.35	25.07	28.29	22.98	24.12	27.17	31.37	24.95	30.65	24.20
AD5	TH2	31.23	25.30	25.09	30.01	27.57	20.04	25.53	29.51	22.95	26.72	33.32	32.88	30.32	30.47	25.52
AD6	KGM	32.65	25.57	26.88	31.74	27.46	19.69	23.35	30.77	23.19	25.26	28.97	34.55	30.61	30.12	25.94
AD6	TH1	28.60	24.91	26.13	22.88	28.06	19.89	23.90	28.49	23.86	23.80	26.32	33.71	26.97	31.11	24.91
AD6	TH2	30.23	25.12	27.40	32.64	28.07	20.86	25.83	n/a	23.48	25.25	30.38	n/a	30.38	31.59	26.64
AD7	KGM	32.08	n/a	26.24	31.27	29.46	19.60	23.42	31.55	22.73	26.02	31.28	32.26	29.78	30.21	26.35
AD7	TH1	28.12	25.29	25.71	22.01	28.07	19.33	25.34	29.37	22.80	23.91	26.59	31.64	25.87	31.02	25.28
AD7	TH2	31.13	25.77	26.40	32.10	28.61	20.90	24.66	32.85	22.50	27.16	33.46	34.32	30.76	30.18	26.49

DIAG	STIM	IL8 (1)	IVL (1)	JUNB (1)	KLK13 (1)	KLK6 (2)	KLK7 (1)	KRT10 (1)	KRT14 (1)	KRT17 (1)	KRT6A (1)	LCN2 (1)	LGMN (1)	MT2A (1)	NELL2 (1)	PI3 (1)
NS1	KGM	36.55	25.16	25.72	30.16	29.28	21.04	20.57	19.49	19.70	18.62	24.88	27.15	23.40	32.87	20.91
NS1	TH1	n/a	27.41	25.63	30.59	27.37	22.74	21.84	20.14	20.65	18.90	22.59	26.43	22.20	33.76	17.92
NS1	TH2	35.83	27.69	24.86	29.71	28.65	21.11	20.64	18.95	20.11	18.66	25.06	27.48	25.12	30.75	21.92
NS2	KGM	32.31	25.86	25.72	30.06	29.91	21.55	19.03	18.68	20.25	18.13	24.88	26.10	23.03	32.40	20.78
NS2	TH1	28.91	26.09	29.09	29.93	28.58	22.39	20.89	19.35	20.67	18.63	21.85	26.52	21.24	32.67	17.39
NS2	TH2	35.15	30.04	24.59	34.25	28.82	24.85	20.38	20.09	22.32	19.72	26.87	27.84	26.76	35.20	22.97
NS3	KGM	29.87	24.31	24.29	27.83	25.88	20.70	20.75	18.18	18.63	17.34	22.70	26.29	21.09	31.98	18.57
NS3	TH1	n/a	25.54	25.32	29.45	27.25	22.19	19.47	18.82	19.27	17.37	21.08	25.64	20.98	33.63	16.44
NS3	TH2	33.46	26.99	24.84	29.44	28.62	21.75	18.55	18.09	19.61	17.08	24.65	26.73	24.68	30.65	19.06
NS4	KGM	34.85	27.22	26.00	31.76	29.38	23.28	19.79	19.29	20.55	19.37	24.79	27.98	24.65	35.22	21.23
NS4	TH1	28.63	25.05	24.75	31.25	27.22	22.79	21.61	19.88	20.69	18.93	21.71	26.05	21.47	33.94	17.01
NS4	TH2	33.28	27.35	24.36	30.20	29.46	21.54	19.15	18.64	19.69	17.89	23.25	26.75	23.33	30.86	19.81
NS5	TH1	28.23	24.39	24.77	30.61	26.37	22.72	22.03	19.60	19.66	18.04	20.91	25.82	20.25	31.02	16.56
NS5	TH2	33.11	25.94	24.72	30.00	27.67	21.24	20.97	18.79	19.01	17.75	23.39	26.66	23.51	29.80	19.72
NS6	KGM	28.17	24.97	25.22	31.09	29.98	24.31	22.07	18.16	19.66	17.14	24.57	25.54	21.66	32.99	19.87
NS6	TH1	25.78	25.62	25.70	31.40	29.95	25.70	23.21	19.18	20.20	18.31	22.41	24.84	21.29	n/a	17.65
NS6	TH2	28.61	28.38	25.28	32.90	32.70	24.31	21.29	18.47	19.68	17.52	26.43	25.98	24.64	33.17	21.82
NS7	KGM	34.62	25.44	25.00	31.13	29.20	24.81	20.14	18.63	20.31	18.09	26.31	25.78	24.10	31.94	21.08
NS7	TH1	26.72	24.92	25.30	31.96	27.12	24.58	23.07	19.54	20.87	18.46	22.35	24.72	20.81	31.97	17.38
NS7	TH2	30.77	27.37	25.80	31.65	29.26	26.91	20.62	18.16	19.30	17.16	26.45	25.35	23.46	32.23	21.59
PS1	KGM	30.98	24.27	24.42	29.82	28.61	21.56	20.50	17.99	18.57	17.20	24.44	25.61	20.79	33.19	18.09
PS1	TH1	28.22	24.42	23.74	29.52	26.68	22.06	20.82	18.03	19.39	17.42	21.80	24.38	18.83	33.34	15.20
PS1	TH2	29.83	25.86	24.81	30.21	29.22	21.97	21.23	18.66	19.83	17.68	25.25	26.07	21.22	29.92	18.57
PS2	KGM	31.81	24.84	24.25	28.33	27.45	21.23	20.89	18.03	17.93	17.17	24.67	n/a	21.96	33.08	18.40
PS2	TH1	28.82	24.40	23.87	27.84	26.15	20.76	22.78	19.02	19.21	17.69	22.54	24.98	20.78	33.92	16.60
PS2	TH2	32.58	26.37	23.94	28.67	27.98	21.67	21.58	18.06	18.66	17.45	25.02	25.35	22.86	31.24	18.59
PS3	KGM	26.59	24.07	24.40	29.77	27.49	22.42	18.59	17.83	18.96	17.05	23.09	25.54	20.94	31.25	18.03
PS3	TH1	29.00	24.64	23.95	29.54	28.63	21.75	20.48	18.98	20.05	18.20	21.56	25.24	20.81	32.56	16.41
PS3	TH2	28.88	25.65	23.74	28.77	29.16	20.71	18.40	17.59	20.12	17.13	24.72	25.69	22.66	27.70	18.91
PS4	KGM	30.54	25.07	24.26	27.27	27.77	20.28	19.48	18.18	19.52	17.22	23.85	25.60	21.12	31.30	19.93
PS4	TH1	26.58	24.83	25.01	28.40	25.14	22.02	20.88	18.63	20.05	17.67	21.19	23.82	19.58	33.49	16.27
PS4	TH2	32.41	27.11	25.16	29.70	28.32	21.65	20.31	18.29	19.60	17.26	24.66	25.75	23.11	29.50	20.75
PS5	KGM	30.81	24.94	24.75	29.74	28.61	21.96	19.91	18.04	20.14	17.32	26.20	25.62	21.71	32.67	20.18
PS5	TH1	26.94	23.82	24.63	29.52	26.91	22.65	20.32	17.76	19.69	17.10	22.88	23.98	20.26	33.65	16.57
PS5	TH2	31.36	26.67	25.51	30.25	28.62	22.23	20.83	17.98	20.08	17.59	27.96	25.58	22.82	30.82	19.82
PS6	KGM	29.46	24.63	26.40	28.97	27.09	22.16	24.58	18.27	20.63	17.87	23.94	24.29	18.80	34.06	18.49
PS6	TH1	25.56	24.51	25.36	28.83	26.43	22.63	22.18	18.38	20.21	18.01	23.02	23.68	19.76	34.71	17.48
PS6	TH2	31.48	26.26	25.91	30.29	27.66	23.57	21.92	18.15	19.74	17.41	25.54	25.03	34.76	30.86	18.84
PS7	KGM	26.58	24.22	25.14	29.21	28.77	23.08	20.99	18.14	20.04	17.65	25.52	25.51	20.17	33.15	19.08
PS7	TH1	25.12	24.84	25.16	30.02	25.96	23.92	21.99	18.94	20.90	18.34	22.62	24.95	19.58	33.73	16.18
PS7	TH2	26.75	26.72	24.75	31.34	29.99	24.59	21.48	18.12	20.60	18.03	27.02	25.73	22.71	30.31	20.00
AD1	KGM	31.81	27.04	24.91	30.44	30.55	23.57	18.61	17.68	19.30	16.93	25.14	26.18	23.99	29.78	21.33
AD1	TH1	29.59	26.35	25.12	31.52	29.81	24.68	20.87	18.54	19.80	17.68	23.39	25.74	21.14	31.20	19.23
AD1	TH2	31.61	27.92	24.62	31.49	30.06	23.26	19.30	17.42	19.70	16.94	25.47	25.75	20.75	27.56	21.34
AD2	KGM	32.62	24.80	24.79	30.11	30.87	22.21	20.23	18.12	18.37	17.23	24.81	25.33	22.82	31.08	20.19
AD2	TH1	29.17	23.79	24.27	30.12	29.31	23.10	20.95	18.35	19.47	17.33	22.66	25.74	20.83	31.92	19.91
AD2	TH2	30.95	26.70	24.83	30.36	28.77	21.86	20.79	18.10	19.43	17.36	25.69	25.72	24.25	28.61	16.63
AD3	KGM	31.98	25.04	24.92	29.69	27.62	22.56	18.73	18.17	19.85	17.61	24.95	25.75	21.93	29.82	20.31
AD3	TH1	28.84	25.50	24.74	29.79	27.12	24.16	20.03	18.68	20.19	18.25	23.05	25.80	20.56	29.63	18.26
AD3	TH2	31.85	26.67	24.86	30.62	28.45	23.16	19.48	18.09	19.84	17.77	25.02	25.76	23.30	27.42	21.23
AD4	KGM	31.37	25.76	25.20	29.98	28.21	23.63	24.58	18.73	19.29	18.20	24.69	25.69	20.01	34.31	22.60
AD4	TH1	30.42	26.45	25.55	31.06	28.80	24.88	21.86	18.72	20.04	19.28	24.20	24.96	21.86	33.78	17.99
AD4	TH2	31.82	27.71	25.14	31.22	30.74	23.85	21.73	17.98	19.56	17.73	27.83	26.01	22.87	29.36	19.73
AD5	KGM	32.15	26.22	25.11	30.59	30.16	23.55	18.82	18.19	19.93	17.72	26.44	26.32	24.30	31.95	21.13
AD5	TH1	29.42	24.92	24.13	29.91	29.39	23.40	20.40	18.41	19.12	17.56	22.62	25.64	20.81	31.37	18.20
AD5	TH2	32.07	27.52	24.04	29.80	29.36	24.24	19.18	17.97	19.92	17.40	25.73	26.38	24.57	28.40	20.96
AD6	KGM	30.99	25.72	25.12	31.05	29.10	22.83	19.67	17.89	18.85	17.20	25.11	25.76	21.88	30.99	21.26
AD6	TH1	29.36	25.32	24.80	30.81	28.22	24.82	21.48	18.87	19.96	17.91	23.61	24.99	20.27	31.42	18.79
AD6	TH2	31.94	27.61	25.02	30.97	30.13	24.62	21.09	18.89	19.79	17.77	26.35	26.10	23.57	28.23	21.66
AD7	KGM	35.26	25.64	25.57	30.39	29.83	23.54	19.05	18.82	20.15	18.27	26.23	26.11	24.06	33.84	22.02
AD7	TH1	30.26	25.38	24.21	30.87	28.90	23.97	20.88	18.71	19.82	17.94	22.69	25.77	21.44	33.12	18.69
AD7	TH2	31.73	27.67	24.72	30.97	31.26	23.62	19.65	18.24	20.17	17.87	27.15	25.41	n/a	30.27	22.45

DIAG	STIM	S100A8 (1)	S100A9 (1)	SLPI (1)	SPRR2C (2)	TGFA (1)	TGM1 (1)	TLR2 (1)	TLR3 (1)	TNC (2)	TNF (1)	TNFRSF6 (1)	TP73L (1)
NS1	KGM	23.24	22.61	17.75	21.39	27.89	26.50	31.24	32.47	32.41	34.88	30.36	23.75
NS1	TH1	19.97	19.64	17.75	19.95	28.65	25.05	32.38	33.44	32.29	33.01	31.09	24.17
NS1	TH2	24.05	22.07	19.34	21.26	27.09	26.93	31.32	29.61	28.35	33.95	27.93	22.86
NS2	KGM	23.05	21.85	18.80	23.91	27.96	27.38	30.28	31.98	32.73	34.14	29.87	23.23
NS2	TH1	17.65	18.00	17.87	20.89	27.81	25.28	29.24	31.53	31.57	28.99	29.41	23.08
NS2	TH2	24.52	25.43	20.74	23.73	31.60	30.41	31.00	32.94	27.71	n/a	29.79	25.45
NS3	KGM	21.93	20.61	16.87	18.88	26.28	24.62	32.78	32.97	30.85	32.76	30.80	23.28
NS3	TH1	17.33	17.79	16.63	18.94	26.59	24.55	30.10	31.61	32.23	30.16	29.16	24.06
NS3	TH2	21.87	22.86	17.74	20.70	27.78	26.30	30.21	31.62	29.74	32.20	29.21	23.09
NS4	KGM	23.13	23.02	17.43	23.74	30.10	27.74	31.26	34.31	32.70	35.04	31.91	24.80
NS4	TH1	18.24	18.57	18.85	20.44	27.17	25.34	28.85	31.58	32.70	29.43	30.51	24.47
NS4	TH2	21.76	22.03	17.86	23.83	28.42	27.31	29.48	31.81	29.72	32.21	29.94	22.71
NS5	TH1	18.10	17.89	17.29	19.15	24.82	23.63	29.25	29.50	28.28	28.97	29.15	24.01
NS5	TH2	22.59	22.44	17.90	21.30	23.48	25.62	29.74	28.17	26.64	30.18	28.92	22.76
NS6	KGM	19.26	19.97	18.98	24.22	25.15	25.90	28.48	29.20	28.81	28.14	29.35	24.00
NS6	TH1	17.29	17.68	18.78	22.38	26.34	26.16	26.83	30.47	26.36	28.17	29.94	23.41
NS6	TH2	21.20	22.60	20.17	26.67	27.43	27.17	28.60	29.83	24.43	32.59	29.39	23.49
NS7	KGM	20.20	21.39	19.87	25.08	26.60	26.47	30.64	29.80	29.63	32.34	30.30	24.67
NS7	TH1	17.59	18.01	18.74	20.58	26.51	25.12	28.79	30.01	27.73	28.84	30.28	23.49
NS7	TH2	20.50	22.47	20.56	25.28	26.85	26.41	28.77	29.78	22.47	29.88	29.04	24.66
PS1	KGM	22.03	21.08	17.01	19.90	24.87	25.01	31.90	34.69	28.59	31.05	29.15	23.09
PS1	TH1	18.14	18.59	16.10	18.42	24.15	23.90	28.70	29.74	25.78	28.56	29.15	23.46
PS1	TH2	22.19	22.18	16.96	20.65	25.57	26.08	29.20	30.62	25.66	32.01	28.91	23.31
PS2	KGM	22.55	22.05	16.71	19.84	25.63	24.43	31.91	31.64	26.01	29.86	29.26	22.67
PS2	TH1	19.64	19.10	16.62	18.47	25.54	23.85	31.49	30.64	26.12	29.96	29.59	23.30
PS2	TH2	23.63	23.49	17.30	20.14	24.61	25.54	30.59	29.68	23.12	31.36	29.42	22.30
PS3	KGM	19.25	19.79	16.41	20.31	25.59	25.01	29.03	30.32	29.81	29.92	28.80	23.20
PS3	TH1	17.53	17.15	15.74	19.27	24.30	24.81	27.63	28.42	26.70	27.13	29.00	23.20
PS3	TH2	22.92	22.99	16.71	21.97	26.35	25.90	28.03	29.75	25.92	29.33	27.67	22.63
PS4	KGM	22.70	22.37	17.99	20.78	25.53	26.27	30.05	30.73	23.36	30.41	29.45	22.90
PS4	TH1	17.94	18.34	17.08	19.12	25.38	25.15	27.46	31.59	23.90	26.38	30.10	24.29
PS4	TH2	23.86	24.24	19.09	21.68	28.29	26.94	30.30	31.10	25.58	32.20	30.11	23.17
PS5	KGM	21.61	22.18	18.07	20.45	27.21	26.44	30.99	31.58	29.11	32.07	29.49	23.46
PS5	TH1	17.38	17.83	16.59	19.34	25.70	24.92	28.00	29.70	28.89	25.94	29.26	23.38
PS5	TH2	21.79	23.38	18.20	21.43	27.00	26.68	29.51	31.78	24.93	29.60	n/a	23.58
PS6	KGM	21.80	21.62	18.64	21.01	26.09	26.85	29.02	32.70	22.30	29.75	29.74	24.23
PS6	TH1	18.61	19.18	18.47	20.63	26.51	26.41	27.14	29.79	29.06	27.74	29.34	23.37
PS6	TH2	21.27	22.41	19.52	22.17	27.60	27.31	28.25	31.88	24.18	31.32	28.99	23.25
PS7	KGM	18.97	19.83	18.17	20.83	27.14	26.25	30.22	31.15	26.02	30.11	29.22	23.32
PS7	TH1	17.59	18.01	17.27	19.58	26.95	25.72	28.66	30.86	25.92	27.31	29.73	24.63
PS7	TH2	20.37	21.67	18.96	22.52	28.32	26.64	30.30	32.25	25.57	29.62	29.71	24.25
AD1	KGM	23.12	24.43	19.05	22.96	26.88	27.46	29.14	31.91	29.03	31.33	30.15	23.40
AD1	TH1	18.24	19.62	18.23	21.82	26.73	26.84	26.88	30.50	26.09	27.35	29.31	24.01
AD1	TH2	23.70	24.35	19.30	23.58	27.40	28.09	27.07	29.77	26.11	30.92	29.27	22.82
AD2	KGM	21.84	22.82	17.66	21.16	27.77	25.68	31.84	33.43	25.36	30.37	29.31	23.00
AD2	TH1	18.05	18.84	17.77	20.37	25.64	24.67	29.16	30.22	25.64	27.20	29.08	23.58
AD2	TH2	23.29	23.89	16.60	21.48	27.49	25.90	28.06	30.87	23.54	29.75	30.19	22.92
AD3	KGM	23.13	24.07	18.78	23.03	26.35	26.33	27.74	32.22	27.88	30.44	29.67	22.95
AD3	TH1	16.82	17.80	18.70	22.32	26.81	26.35	25.93	29.59	23.21	25.98	29.48	23.57
AD3	TH2	23.34	25.22	19.27	23.50	27.88	27.15	26.98	30.65	23.59	29.83	29.90	23.50
AD4	KGM	23.20	23.18	18.22	21.69	26.48	26.85	29.88	32.61	24.54	27.78	29.51	23.85
AD4	TH1	18.88	20.44	18.10	21.40	26.75	26.79	28.16	28.85	24.67	27.35	29.42	23.99
AD4	TH2	23.28	24.85	19.53	23.01	29.42	28.20	30.68	33.11	26.14	30.69	30.07	22.86
AD5	KGM	22.90	24.59	18.71	22.13	28.97	27.90	31.87	33.15	30.02	33.03	33.58	23.92
AD5	TH1	17.31	17.97	17.42	21.07	26.45	24.80	28.60	29.10	28.56	26.81	28.60	22.95
AD5	TH2	23.98	24.84	19.05	22.51	27.04	27.72	28.43	30.67	27.30	30.50	28.96	22.66
AD6	KGM	21.13	22.70	19.18	23.02	28.08	27.59	27.93	31.53	25.64	31.09	29.93	23.30
AD6	TH1	17.96	18.45	18.78	21.91	26.90	26.98	27.10	29.62	26.28	27.10	29.53	25.26
AD6	TH2	22.38	24.76	20.28	23.46	27.68	29.19	28.02	31.29	25.65	29.79	29.21	23.37
AD7	KGM	22.40	23.66	18.84	22.44	27.73	27.03	31.64	30.20	27.59	30.64	29.74	24.52
AD7	TH1	16.84	18.29	17.68	20.27	26.68	24.86	30.39	29.50	27.78	30.19	29.08	23.59
AD7	TH2	23.82	25.06	19.60	24.16	27.73	28.10	29.19	30.63	28.03	26.94	28.82	23.50
