# Supplementary material for: Genetically Programmed Differences in Epidermal Host Defense between Psoriasis and Atopic Dermatitis Patients
Source: PLoS One. 2008 Jun 4;3(6):e2301. doi: 10.1371/journal.pone.0002301 (PMC2409155; doi:10.1371/journal.pone.0002301)
Supplement: Table S3 — least square means of ΔCt values of each gene for all diagnoses and stimuli; fold increase of stimuli and diagnoses (0.08 MB PDF) [file pone.0002301.s003.pdf]

Table S3. **Least square means of ΔCt values of each gene for all diagnoses and stimuli; fold increase of stimuli and diagnoses**

	least square means of ΔCt			least square means of ΔCt			fold increase				
**GENE**	**NS**	**PS**	**AD**	**KGM**	**Th1**	**Th2**	**PS/NS**	**AD/NS**	**PS/AD**	**Th1/KGM**	**Th2/KGM**
DEFB103	8.9	4.5	7.8	7.6	5.8	7.9	21.3	2.1	9.9	3.4	0.8
S100A9	3.1	2.6	5.6	4.8	0.4	6.2	1.4	0.2	8.1	20.4	0.4
S100A8	2.5	2.4	4.9	4.5	0.0	5.4	1.1	0.2	5.9	23.7	0.6
DEFB4	10.0	6.7	11.2	12.0	2.8	13.1	10.1	0.5	22.1	591.3	0.5
CST6	6.7	4.7	5.8	6.0	4.6	6.6	4.0	1.8	2.2	2.7	0.7
LCN2	6.3	6.6	7.4	7.4	4.4	8.4	0.8	0.5	1.8	8.1	0.5
IL1F9	9.9	10.8	12.3	12.5	7.8	12.7	0.5	0.2	2.7	27.1	0.9
CXCL10	12.6	12.5	14.9	16.0	8.5	15.6	1.0	0.2	5.3	187.7	1.3
SPRR2C	4.7	2.3	4.0	4.0	2.0	5.0	5.2	1.5	3.4	4.0	0.5
CTSL2	3.6	4.6	5.5	4.4	3.7	5.6	0.5	0.3	1.9	1.6	0.4
CCL20	10.2	10.2	12.4	12.3	8.5	12.0	1.0	0.2	4.4	13.4	1.2
IVL	8.0	7.6	9.1	7.8	7.2	9.8	1.3	0.5	2.8	1.5	0.2
IL1B	8.7	10.0	13.5	10.8	8.3	13.2	0.4	0.0	11.4	5.7	0.2
CXCL2	11.7	10.8	13.7	12.9	9.9	13.5	1.8	0.2	7.5	7.9	0.6
CCL5	11.0	9.6	11.9	12.2	8.8	11.6	2.6	0.5	4.9	10.6	1.5
CA2	2.5	1.9	1.8	2.8	2.8	0.6	1.5	1.5	1.0	1.0	4.6
CALML5	7.3	8.6	9.1	8.6	7.5	8.9	0.4	0.3	1.5	2.2	0.8
LGMN	7.6	8.0	8.9	8.6	7.2	8.7	0.8	0.4	1.8	2.5	0.9
TGM1	8.0	8.5	9.5	9.0	7.3	9.8	0.7	0.4	2.0	3.2	0.6
TLR2	11.0	11.7	12.9	13.1	10.6	11.9	0.7	0.3	2.3	5.4	2.2
SLPI	1.1	-0.2	0.9	0.8	-0.3	1.5	2.5	1.2	2.1	2.2	0.6
CTSL	8.8	8.3	9.6	9.1	8.3	9.1	1.4	0.6	2.4	1.8	1.0
PI3	1.6	0.3	3.4	2.8	-0.6	3.0	2.5	0.3	8.9	10.2	0.9
IL1A	6.9	7.8	8.4	8.1	6.3	8.7	0.6	0.4	1.6	3.4	0.6
IL1RN	7.1	7.1	8.5	7.8	6.5	8.5	1.0	0.4	2.7	2.5	0.6
IL8	13.5	9.2	16.0	14.1	10.3	14.4	19.2	0.2	110.2	14.2	0.8
KLK6	9.9	9.9	12.1	10.9	9.4	11.7	1.0	0.2	4.6	2.9	0.6
NELL2	13.6	14.2	14.7	14.9	14.8	12.8	0.6	0.4	1.4	1.1	4.2
CCL27	12.7	10.4	11.1	12.7	9.3	12.1	4.9	3.1	1.6	10.7	1.5
MT2A	4.7	3.0	5.9	4.8	2.7	6.1	3.2	0.4	7.5	4.2	0.4
IL1F8	15.7	14.5	15.3	15.5	13.5	16.4	2.2	1.3	1.7	4.1	0.5
TNF	13.0	12.0	12.7	13.9	10.2	13.5	2.1	1.2	1.7	13.0	1.3
KLK7	6.0	4.8	5.1	5.0	5.2	5.7	2.4	1.9	1.3	0.9	0.6
FABP5	2.6	2.7	2.9	2.6	2.1	3.4	0.9	0.8	1.1	1.4	0.6
IL18	4.7	4.6	5.1	4.6	4.8	5.0	1.1	0.8	1.4	0.8	0.8
Arg1	12.9	13.7	13.5	13.5	12.3	14.3	0.6	0.7	0.9	2.3	0.6
TGFA	8.4	9.5	9.9	9.4	8.3	10.1	0.5	0.3	1.3	2.2	0.6
TNC	8.8	8.4	10.8	10.4	9.3	8.3	1.4	0.3	5.3	2.2	4.3
KLK13	13.0	11.9	13.0	12.4	12.2	13.3	2.1	1.0	2.1	1.2	0.6
TLR3	12.5	14.3	13.5	14.5	12.3	13.5	0.3	0.5	0.6	4.5	1.9
IER3	11.3	11.4	14.1	12.8	11.0	13.0	0.9	0.1	6.3	3.5	0.8
AREG	5.6	5.1	5.9	5.5	5.0	6.2	1.4	0.8	1.7	1.4	0.6
KRT10	3.2	3.7	2.6	3.0	3.3	3.1	0.7	1.5	0.5	0.8	1.0
DKK1	10.8	9.2	10.0	10.1	9.6	10.4	3.2	1.8	1.8	1.4	0.8
CXCL1	10.5	7.1	11.6	10.5	7.7	11.0	10.3	0.5	21.8	7.1	0.7
GJA1	6.8	7.4	8.0	7.5	7.1	7.5	0.6	0.4	1.5	1.3	1.0
TNFRSF6	11.9	11.9	12.1	12.4	11.5	11.9	1.0	0.9	1.1	1.9	1.4
CAMP	14.7	13.6	14.0	14.2	13.6	14.5	2.2	1.7	1.3	1.4	0.8
KRT6A	-0.2	0.2	0.7	0.3	0.1	0.3	0.8	0.5	1.5	1.2	1.0
DEFB1	8.3	7.9	8.2	8.4	7.4	8.5	1.3	1.1	1.3	1.9	0.9
KRT17	1.8	2.4	2.5	2.2	2.0	2.5	0.6	0.6	1.0	1.1	0.8
IL1R1	13.8	12.7	13.9	13.4	13.6	13.4	2.2	0.9	2.4	0.9	1.0
KRT14	0.8	0.7	1.4	1.0	0.9	1.0	1.1	0.6	1.6	1.1	1.0
JUNB	7.3	7.2	7.6	7.6	7.0	7.5	1.0	0.8	1.3	1.5	1.1
TP73L	5.8	5.9	6.2	6.2	5.8	6.0	0.9	0.8	1.2	1.3	1.1

Least square means of the ΔCt values for each diagnosis (over all stimuli) and for each stimulus (over all diagnoses) are given. Fold increase in gene expression is given as calculated by the method of Livak et al. 2001, based on the least square means. Fold increases or decreases of >4 are marked in red.
