# Supplementary material for: Genetically Programmed Differences in Epidermal Host Defense between Psoriasis and Atopic Dermatitis Patients
Source: PLoS One. 2008 Jun 4;3(6):e2301. doi: 10.1371/journal.pone.0002301 (PMC2409155; doi:10.1371/journal.pone.0002301)
Supplement: Table S4 — protein data on 6 genes for all cultures (0.07 MB PDF) [file pone.0002301.s004.pdf]

Table S4. protein data of 6 genes for all cultures

RANTES and IP-10 were determined by fluoresent bead assay
hBD-2 , SLPI and Elafin were determined by ELISA

detection limits	RANTES	2 pg/ml
	hBD-2	0.5 ng/ml
	CXCL8	30 pg/ml
	IP-10	3 pg/ml
	Elafin	2 ng/ml
	SLPI	2 ng/ml

Samples that were below the detection limit were given the value of the respective detetection limit

sample	diagnosis	treatment	RANTES pg/ml	hBD-2 ng/ml	CXCL8 pg/ml	IP-10 pg/ml	Elafin ng/ml	SLPI ng/ml
1	AD	KGM	159	0.5	532	144	92	52
2	AD	KGM	95	0.5	30	444	444	172
3	AD	KGM	137	0.5	19	302	127	77
4	AD	KGM	60	0.5	290	124	378	44
5	AD	KGM	72	0.5	30	682	30	153
6	AD	KGM	78	0.5	92	258	111	60
7	AD	KGM	66	0.5	30	438	85	91
8	AD	TH1	10913	0.5	1303	751254	132	36
9	AD	TH1	3729	7.2	1983	1156572	1334	277
10	AD	TH1	3613	10.0	3056	694258	280	131
11	AD	TH1	7905	5.2	8809	917916	626	99
12	AD	TH1	2353	6.3	2048	767562	93	108
13	AD	TH1	3343	1.5	3694	731201	195	87
14	AD	TH1	2824	4.1	2412	684954	249	142
15	AD	TH2	56	0.5	748	132	92	61
16	AD	TH2	144	0.5	40	510	508	157
17	AD	TH2	412	0.5	330	388	124	55
18	AD	TH2	148	0.5	117	268	294	57
19	AD	TH2	198	0.5	30	627	232	91
20	AD	TH2	154	0.5	49	250	30	35
21	AD	TH2	147	0.5	13	326	88	68
22	NS	KGM	23	0.5	30	94	147	192
23	NS	KGM	11	0.5	30	241	97	112
24	NS	KGM	29	0.5	30	139	416	274
25	NS	KGM	26	0.5	30	154	228	237
26	NS	KGM	103	0.5	30	333	249	274
27	NS	KGM	28	0.5	947	1465	182	44
28	NS	KGM	35	0.5	30	1021	98	42
29	NS	TH1	1910	6.5	1730	741868	565	321
30	NS	TH1	6434	9.5	5336	827551	704	166
31	NS	TH1	5738	17.3	3525	901105	1313	479
32	NS	TH1	2158	5.0	4528	1100709	1413	509
33	NS	TH1	6796	9.9	4607	685593	1252	378
34	NS	TH1	6888	5.3	28152	819416	490	62
35	NS	TH1	15115	8.7	15949	716616	391	100
36	NS	TH2	55	0.5	30	183	135	143
37	NS	TH2	24	0.5	30	186	85	92
38	NS	TH2	85	0.5	33	284	457	270
39	NS	TH2	50	0.5	30	213	302	161
40	NS	TH2	78	0.5	30	102	395	234
41	NS	TH2	37	0.5	348	2121	112	29
42	NS	TH2	50	0.5	35	1502	93	29
43	PS	KGM	53	0.5	114	446	400	181
44	PS	KGM	87	0.5	179	651	397	146
45	PS	KGM	86	0.5	34	369	644	219
46	PS	KGM	32	0.5	73	145	124	80
47	PS	KGM	123	0.5	106	600	157	116
48	PS	KGM	158	0.5	545	144	200	43
49	PS	KGM	200	0.5	2410	512	202	115
50	PS	TH1	6888	10.8	6283	1480338	1376	290
51	PS	TH1	3968	13.4	3321	668409	950	306
52	PS	TH1	3065	8.0	991	892776	2065	291
53	PS	TH1	8144	7.4	14176	977495	597	178
54	PS	TH1	13152	11.4	4505	1186730	347	205
55	PS	TH1	13942	8.2	6798	735671	294	64
56	PS	TH1	15358	22.6	87777	901555	795	242
57	PS	TH2	102	0.5	122	183	431	163
58	PS	TH2	126	0.5	170	219	446	103
59	PS	TH2	215	0.5	30	362	458	164
60	PS	TH2	51	0.5	87	265	114	65
61	PS	TH2	222	0.5	123	942	149	83
62	PS	TH2	171	0.5	319	650	206	51
63	PS	TH2	332	0.5	2404	618	188	95
