# Supplementary material for: Genetically Programmed Differences in Epidermal Host Defense between Psoriasis and Atopic Dermatitis Patients
Source: PLoS One. 2008 Jun 4;3(6):e2301. doi: 10.1371/journal.pone.0002301 (PMC2409155; doi:10.1371/journal.pone.0002301)
Supplement: Table S5 — p-values of post-hoc test on protein data (0.06 MB PDF) [file pone.0002301.s005.pdf]

Table S5. p-values of post-hoc test on protein data

	p-value			p-values PS vs NS			p-values AD vs NS			p-values PS vs AD			p-values all diagnoses	
Protein	Diagnosis	Stimulus	Cross effect	KGM	Th1	Th2	KGM	Th1	Th2	KGM	Th1	Th2	Th1 vs KGM	Th2 vs KGM
CXCL8	0.0235	0.0000	0.5142	0.0736	0.8351	0.0616	0.6063	0.2326	0.4063	0.1732	0.1874	0.2359	0.0001	0.9939
hBD-2	0.0110	0.0000	0.0019	1.0000	0.1883	1.0000	1.0000	0.0004	1.0000	1.0000	0.0001	1.0000	0.0001	1.0000
elafin	0.0046	0.0000	0.6778	0.3963	0.9118	0.4262	0.4147	0.0155	0.5333	0.1160	0.0155	0.1865	0.0001	0.9380
RANTES	0.0000	0.0000	0.0281	0.0014	0.1984	0.0019	0.0012	0.5214	0.0016	0.9724	0.0701	0.9202	0.0001	0.0052
SLPI	0.0240	0.0051	0.9531	0.6901	0.7666	0.8583	0.2466	0.0635	0.2998	0.4082	0.1034	0.3634	0.0269	0.2921
IP-10	0.6505	0.0000	0.9999	0.7069	0.6840	0.7701	0.9161	0.9551	0.8040	0.6464	0.6646	0.6138	0.0001	0.5766

Analysis of ELISA and fluorescent bead assays for six secreted proteins of 63 cultures. For raw data see Table S4. Protein data largely corresponded to qPCR data, showing a strong effect of Th1 cytokines on gene expression. The factor diagnosis was significant for most proteins. The p-values of diagnosis, stimulus and cross effect were obtained by factorial ANOVA on log transformed data. Significances of the subgroup comparisons were obtained by post-hoc testing (Duncan's multiple range test). p-values smaller than 0.05 are indicated in red.
