# Supplementary material for: Genetically Programmed Differences in Epidermal Host Defense between Psoriasis and Atopic Dermatitis Patients
Source: PLoS One. 2008 Jun 4;3(6):e2301. doi: 10.1371/journal.pone.0002301 (PMC2409155; doi:10.1371/journal.pone.0002301)
Supplement: Text S1 — statistics and graphical representations (0.01 MB RTF) [file pone.0002301.s006.doc]

Text S1

Statistics and graphical representations
All data were analyzed with the Statistica software package version 7.0 (StatSoft Inc). For the qPCR experiments, statistical analysis was performed on the Ct values, which is the difference between the Ct of the target gene and the reference gene. The general linear models module was used to analyze the data (design: repeated factorial ANOVA). Correction for multiple testing (FDR) was performed as described by Verhoeven et al[1]. Post-hoc testing for significance was done by Duncan's multiple range test. The least square means and standard errors were calculated and used in Table S3 and Figure 4, to obtain a fold difference and graphical representation of the data. The relative expression levels (Figure 4) were obtained as described by Livak and Schmittgen[2]. Please note that this will yield asymmetric error bars because of the conversion of an exponential variable to obtain a linear comparison. For p-values we refer to Figure 3, which are derived from the Ct values.
The multivariate exploratory techniques module was used for cluster analysis. Only those genes that passed the test for false discovery rate (51 out of 55) were included in the analysis. qPCR data were subjected to Z-transformation and the Euclidian distance was used as a dissimilarity measure. Columns and rows were clustered by Ward's amalgamation rule. Sorting in two dimensions reorganizes the data and generates an expression matrix depicted as a heat-map in which each cell was assigned a color corresponding to its normalized value.
For statistical analysis of the protein data, the raw figures obtained from ELISA or fluorescent bead assays were log-transformed and analyzed by factorial ANOVA. Post-hoc testing was performed by Duncan's multiple range test. For graphical representation (Figure 4) we used the protein concentrations as they were read from the calibration curves of the respective assays, and bars are depicted as means with standard deviations.

References

	1. 	Verhoeven KJF, Simonsen KL, McIntyre LM (2005) Implementing false discovery rate control: increasing your power. Oikos 108: 643-647.
	2. 	Livak KJ, Schmittgen TD (2001) Analysis of relative gene expression data using real-time quantitative PCR and the 2(-Delta Delta C(T)) Method. Methods 25: 402-408.
